# Epithelial‐to‐mesenchymal transition (EMT) to sarcoma in recurrent lung adenosquamous carcinoma following adjuvant chemotherapy

**DOI:** 10.1111/1759-7714.13156

**Published:** 2019-07-27

**Authors:** Mau Ern Poh, Chong Kin Liam, Kein Seong Mun, Chee Shee Chai, Chee Kuan Wong, Jiunn Liang Tan, Thian Chee Loh, Ka Kiat Chin

**Affiliations:** ^1^ Department of Medicine, Faculty of Medicine University of Malaya Kuala Lumpur Malaysia; ^2^ Department of Pathology, Faculty of Medicine University of Malaya Kuala Lumpur Malaysia; ^3^ Department of Medicine, Faculty of Medicine University Malaysia Sarawak, Sarawak Malaysia

**Keywords:** Adenosquamous lung carcinoma, adjuvant chemotherapy, epithelial‐to‐mesenchymal transition, lung cancer, sarcoma

## Abstract

Adjuvant chemotherapy has long been indicated to extend survival in completely resected stage IB to IIIA non‐small cell lung cancer (NSCLC). However, there is accumulating evidence that chemotherapy or chemoradiotherapy can induce epithelial‐to‐mesenchymal transition (EMT) in disseminated or circulating NSCLC cells. Here, we describe the first case of EMT as the cause of recurrence and metastasis in a patient with resected stage IIB lung adenosquamous carcinoma after adjuvant chemotherapy. We review the literature and explore the possible mechanisms by which EMT occurs in disseminated tumor cells (DTC) or circulating tumor cells (CTC) in response to adjuvant chemotherapy (cisplatin) as a stressor. We also explore the possible therapeutic strategies to reverse EMT in patients with recurrence. In summary, although adjuvant cisplatin‐based chemotherapy in resected NSCLC does extend survival, it may lead to the adverse phenomenon of EMT in disseminated tumor cells (DTC) or circulating tumor cells (CTC) causing recurrence and metastasis.

## Introduction

There is accumulating evidence that chemotherapy or chemoradiotherapy can induce epithelial‐to‐mesenchymal transition (EMT) in non‐small cell lung cancer cells. Here, we describe the first case of EMT as the cause of recurrence and metastasis in a patient with resected stage IIB lung adenosquamous carcinoma after adjuvant chemotherapy.

### Case report

**Figure 1:**
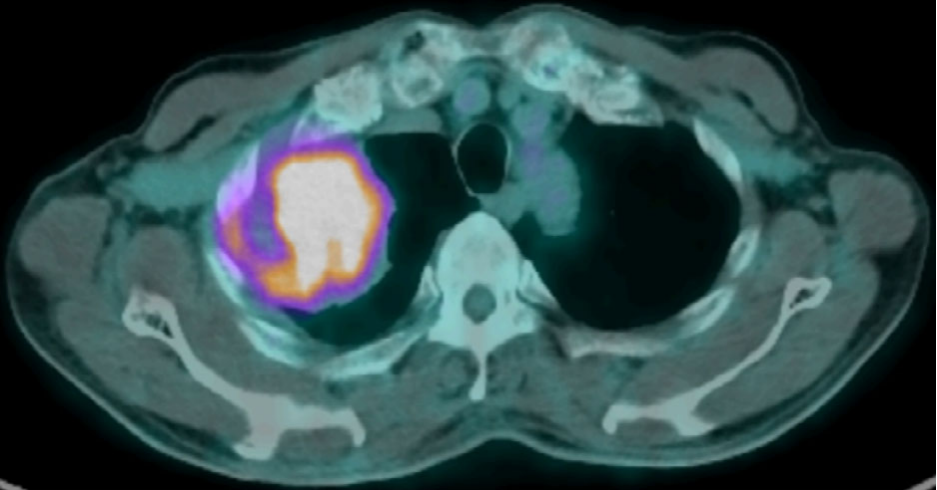
Fluorine‐18 (18F‐) fluorodeoxyglucose (FDG) positron emission tomography (PET)‐CT scan revealed high uptake of 18F‐FDG by the right upper lobe mass (6.8 cm x 6.4 cm x 6.4 cm) with no distant 18F‐FDG avid lesions.

**Figure 2:**
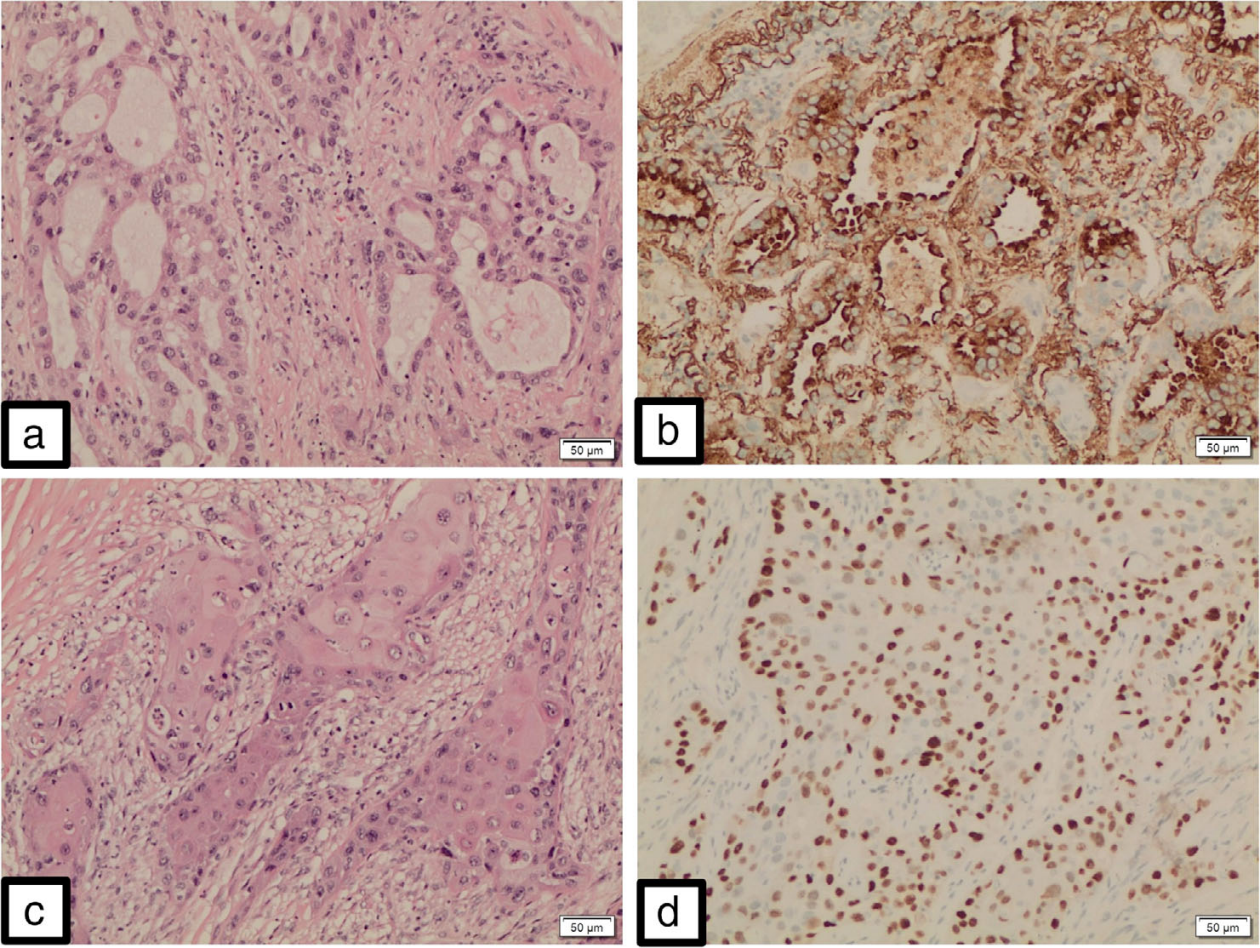
Malignant glandular component made up of dysplastic cells arranged in distinct confluent glandular‐cribriform clusters (**a**). These neoplastic glandular elements are positive for Napsin A (**b**) and negative for p63. Malignant squamous component made up of polygonal tumor cells arranged in solid infiltrative clusters (**c**). Individual cell keratinization and intercellular bridges are clearly evident. These neoplastic squamoid elements are positive for p63 (**d**) and negative for Napsin A. (x100).

**Figure 3:**
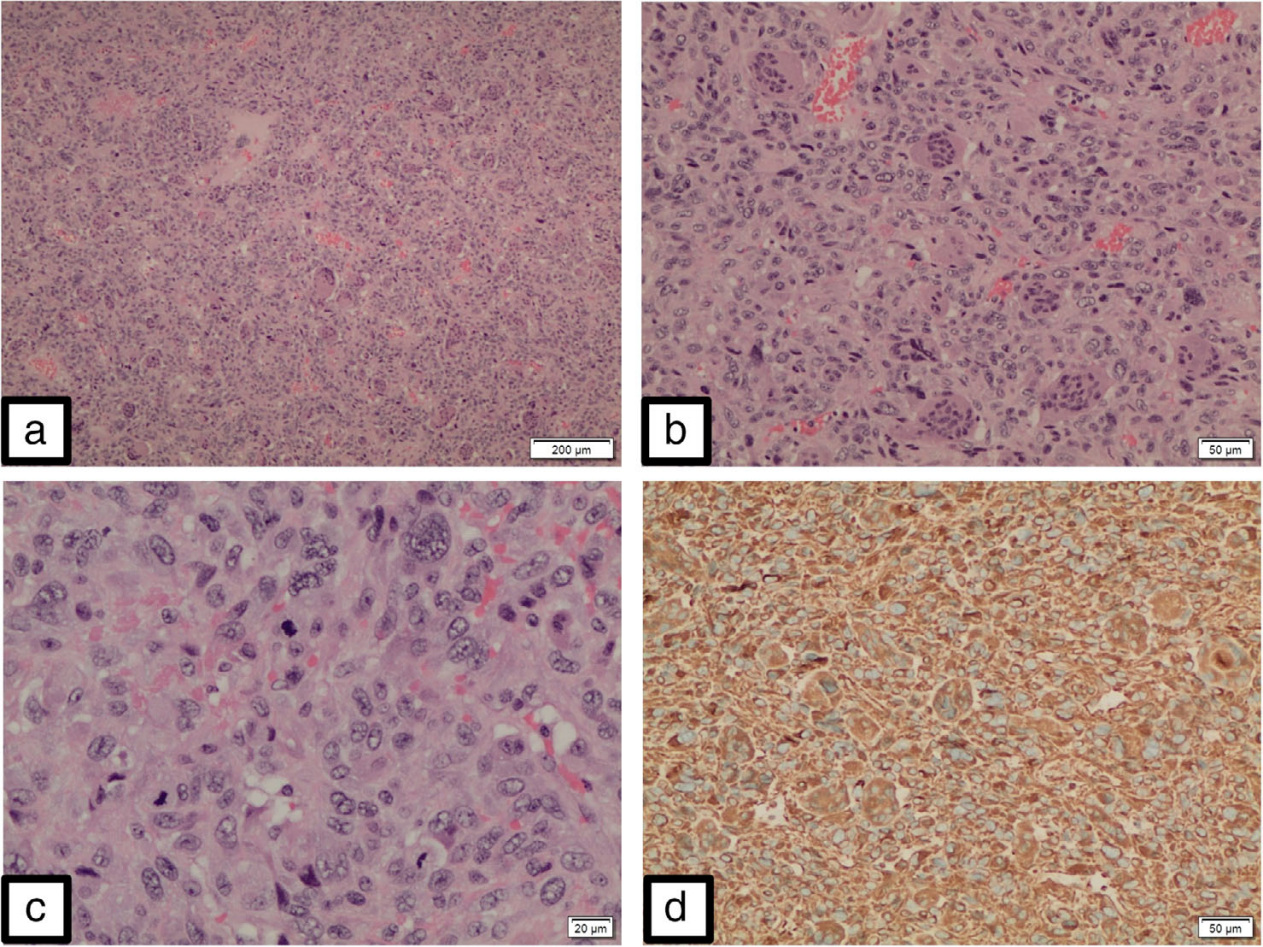
Malignant mesenchymal tumor, i.e., sarcoma, resembling giant cell tumor of bone and soft tissue. This tumor is made up of numerous osteoclastic‐type multinucleated giant cells in a background of malignant mononuclear cells (**a,b**). The mononuclear cells exhibit focal marked pleomorphism and increased mitoses (**c**). The tumor cells are strongly positive for vimentin (**d**), and negative for TTF‐1 and CK5/6. (x100).

A 72‐year‐old man who had never smoked underwent a right upper lobectomy for adenosquamous carcinoma. A preoperative fluorine‐18 fluorodeoxyglucose positron emission tomography‐CT scan did not show any metastasis (Fig 1). The resected specimen showed adenosquamous carcinoma without any sarcomatous component measuring 6.5 cm x 4 cm x 3.5 cm with visceral pleural invasion and lymphovascular permeation (Fig 2). The surgical margins were clear and the resected intrathoracic lymph nodes were free of metastasis (pathological stage IIB [pT3N0M0]). Adjuvant chemotherapy consisted of four cycles of cisplatin 75 mg/m^2^ on day 1 and vinorelbine 25 mg/m^2^ on days 1 and 8 every three weeks. A repeat CT examination eight months post‐surgery showed a recurrent tumor at the apex of the remaining right lung measuring 7.0 cm x 6.6 cm x 3.7 cm. He underwent a surgical resection of the tumor and reconstruction of the chest wall. Histopathological examination of the tumor revealed a high grade pleomorphic sarcoma with no epithelial elements. The tumor cells were strongly positive for vimentin and negative for cytokeratin (CK) 5 and 6, and thyroid transcription factor‐1 (TTF‐1) (Fig 3). A CT scan two months later showed multiple new metastatic lung nodules.

## Discussion

To our knowledge, this is the first reported case of EMT as the cause of recurrence in a patient with resected stage IIB non‐small‐cell lung carcinoma (NSCLC) after adjuvant chemotherapy. EMT has so far been reported to cause acquired resistance to epidermal growth factor receptor (EGFR)‐tyrosine kinase inhibitors.^1^

Numerous studies have been carried out to determine why recurrence develops after complete resection of NSCLC.^2^ Recurrence after complete resection of NSCLC has largely been attributed to micro‐metastatic cancer cells already present systemically at the time of surgery, which are undetected by standard staging methods including modern diagnostic imaging.^3^ Disseminated tumor cells (DTCs) or circulating tumor cells (CTCs) have also been described.^3^ However, it is unclear whether these cells have proliferative activity or are just “dormant cells”. Adjuvant cisplatin‐based chemotherapy has been shown to increase the median survival in patients with completely resected stage IB to IIIA NSCLC, possibly by eliminating the cells described above thus reducing the risk of recurrence and metastasis.^4^

In the process of tumor dissemination or metastasis, some tumor cells acquire new characters, as an expression of mesenchymal markers and loss of epithelial markers, and undergo profound morphogenetic changes, collectively referred to as EMT. EMT confers an invasive phenotype and facilitates the dissemination of cancer cells to distant organs. In addition to facilitating metastasis, EMT is thought to generate cancer stem cells (CSCs), which are generally resistant to apoptosis and to standard chemotherapeutic drugs and radiotherapy.^5^, ^6^, ^7^, ^8^ There is also increasing evidence that treatment with chemotherapy or chemoradiotherapy can induce EMT in NSCLC which in turn is thought to generate CSCs which are generally resistant to such treatments.^9^, ^10^, ^11^, ^12^

EMT activation can be induced by genetic mutations occurring in cancer cells or external environmental stimuli such as chemotherapy. Several mechanisms behind chemotherapy‐induced EMT have been recently described.

Firstly, cisplatin has been shown to increase the release of Interleukin‐6 (IL‐6) and expression of transforming growth factor beta (TGF‐β) from cancer‐associated fibroblasts (CAFs).^13^, ^14^ IL‐6 serves to block apoptosis in cells during the inflammatory process, keeping the cells alive in very toxic environments. Unfortunately, these same pathways also serve to protect cancer cells from cellular apoptotic deletion and chemotherapeutic drugs.^15^, ^16^ IL‐6, which enhances TGF‐β‐induced EMT changes in NSCLC, may contribute to the maintenance of a paracrine loop that functions as part of the communication between CAFs and NSCLC cells, resulting in chemoresistance.^17^

Secondly, the transcription factors of the Snail family have long been associated with EMT and cisplatin resistance during cancer metastasis.^18^ The three members of the Snail family encode zinc finger‐type transcription factors. These have been called Snail (Snail1), Slug (Snail 2) and Smuc (Snail3).^18^ Elevated expression of the Snail family transcription factors have been associated with downregulation of epithelial markers (reduced E‐cadherin expression) and upregulation of mesenchymal markers (Vimentin), thereby inducing EMT and generating CSCs that are resistant to conventional chemotherapy.^18^ They are therefore considered as potent EMT inducers associated with cancer cell dissemination.

Thirdly, aside from TGF‐β and Snail, several other signalling pathways including Notch, Wnt, and integrin are known to activate EMT through transcriptional repression of E‐cadherin.^19^, ^20^, ^21^ Other EMT‐controlling transcription factors including Twist and Zing finger E‐box‐Binding (Zeb) 1/2 also function as molecular switches of the EMT programme, causing downregulation of E‐cadherin.^22^, ^23^, ^24^ These transcriptional factors are important EMT‐inducers and by inducing CSCs‐like features, are a major cause of tumor recurrence, metastases and resistance to chemotherapy and radiotherapy.^25^

Finally, low expressions of miRNA‐17, 20a, 20b have been correlated to activate the TGF‐β signalling pathway and induce EMT by which cells become cisplatin‐resistant and migrate.^26^ A separate study reported that through treatment with cisplatin, IL‐6 secretion is upregulated in lung cancer cells by activating the ataxia‐telangiectasia mutated/NF‐kappaB pathway.^27^ This finding demonstrated that the chemotherapeutic agent itself can potentially increase IL‐6 expression in CTCs or DTCs, hence augmenting anti‐apoptotic protein expressions described above, making them resistant to standard chemotherapy.

Therapeutic strategies to reverse or block EMT in patients with recurrence are complex but promising. These include blocking M2 muscarinic receptor signalling^28^; targeting EMT with histone deacetylase inhibitors such as entinostat^29^ and MEK‐inhibitors; inhibition of microRNAs^26^, ^30^ and fibroblast growth factor receptor‐1^31^; using immunotherapy^32^; notch inhibitors^33^; Connexin43,^34^ MCL‐1^35^; and targeting EMT‐transcription factors such as Snail expression.^22^, ^24^, ^36^

In conclusion, while adjuvant cisplatin‐based chemotherapy has been shown to extend survival in completely resected stage IA to IIIA NSCLC, it may also result in the phenomenon of EMT in disseminated tumor cells (DTC), or circulating tumor cells (CTC) causing recurrence and metastasis. Further investigations to study the contribution of external stimuli such as chemotherapy to tumor microenvironment will lead to a more comprehensive understanding of the role of various transcription factors and anti‐apoptotic expression factors in lung cancer, thus providing clinicians with more effective strategies to prevent and treat recurrent metastatic disease.

## Disclosure

The patient has given written consent for this case to be written and published without any identifying information.

The authors declare that they have no conflicts of interest.
